# Why gender and sex matter in infectious disease modelling: A conceptual framework

**DOI:** 10.1016/j.ssmph.2025.101775

**Published:** 2025-03-12

**Authors:** Diane Auderset, Julien Riou, Carole Clair, Matthieu Perreau, Yolanda Mueller, Joëlle Schwarz

**Affiliations:** a Faculty of Health Sciences, Simon Fraser University, Burnaby, Canada; b Gender and Health Unit, Department of Ambulatory Care, Unisanté, Centre for Primary Care and Public Health & University of Lausanne, Lausanne, Switzerland; c Department of Family Medicine, Unisanté, Centre for Primary Care and Public Health & University of Lausanne, Lausanne, Switzerland; d Department of Epidemiology and Health Systems, Unisanté, Centre for Primary Care and Public Health & University of Lausanne, Lausanne, Switzerland; e Service of Immunology and Allergy, Lausanne University Hospital and University of Lausanne, Lausanne, Switzerland

**Keywords:** Infectious disease modelling, Gender disparities in health outcomes, Sex-based differences, Gender/sex-sensitive modelling, Social epidemiology, COVID-19 transmission dynamics, SEIRD compartmental model

## Abstract

The COVID-19 pandemic underscored the differential impact of infectious diseases across population groups, with gender and sex identified as important dimensions influencing transmission and health outcomes. Sex-related biological factors, such as differences in immune response and comorbidities, contribute to men's heightened severity risks, while gender norms and roles influence exposure patterns, adherence to prevention measures, and healthcare access, influencing women's higher reported infection rates in certain contexts. Despite widely observed gender/sex disparities, infectious disease models frequently overlook gender and sex as key dimensions, leading to gaps in understanding and potential blind spots in public health interventions. This paper develops a conceptual framework based on the Susceptible-Exposed-Infectious-Recovered/Deceased (SEIR/D) compartmental model to map pathways through which gender and sex may influence susceptibility, exposure, transmission, recovery, and mortality. Using a narrative review of modelling, epidemiological, and clinical studies, this framework identifies and characterises the main social and biological mechanisms on this matter—including gendered occupational exposure, differential adherence to preventive measures, and disparities in healthcare-seeking behaviour—alongside sex-based differences in immune response and disease severity. The framework also examines potential gender-related variations in epidemiological surveillance data, highlighting disparities in testing uptake and hospitalisation referrals that could influence model outputs. By synthesising these insights, this paper provides a theoretical foundation for integrating gender and sex into infectious disease models. It advocates for interdisciplinary collaboration between modellers, social scientists, and clinicians to advance gender- and sex-sensitive modelling approaches. Accounting for gender and sex can enhance predictive accuracy, inform intervention strategies, and promote health equity in pandemic response.

## Highlights

• Gender and sex influence COVID-19 transmission, severity, and mortality risks.
• Infectious disease models rarely account for these social and biological factors.
• A conceptual SEIR/D framework maps gender and sex influences on disease progression.
• Integrating gender and sex improves model predictions and public health interventions.

## 1 Introduction

### 1.1 Background*:* mathematical modelling of infectious diseases

Infectious disease transmission models are important tools for understanding transmission dynamics, predicting outbreaks, and guiding public health responses. By linking mechanisms such as exposure, contagion, and immunity, these models quantify key parameters—transmission rates, reproduction numbers, and herd immunity thresholds—to forecast outbreaks and evaluate intervention strategies. Their importance was underscored during the COVID-19 pandemic, where policymakers frequently referenced concepts such as “flattening the curve” to stress the importance of contact reduction to control virus spread. Transmission models shaped public health policies, optimised resource allocation, and guided effective prevention and control measures (Jit & Cook, 2024; Kong et al., 2022). These models provided early projections that informed policymaking, particularly in the United Kingdom (UK) and the United States of America (USA), illustrating the potential impact of non-pharmaceutical interventions (NPIs) like physical distancing, isolation, and lockdowns on reducing mortality and healthcare demand (Ferguson et al., 2020; Kong et al., 2022).

**Fig. 1:**
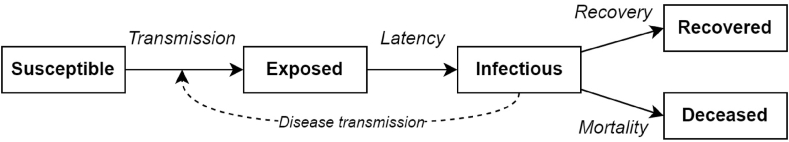
The Susceptible-Exposed-Infected-Recovered/Deceased (SEIRD) model and transition dynamics.

Mechanistic models, particularly compartmental models, simulate disease progression by dividing the population into discrete groups such as Susceptible (S), Exposed (E), Infectious (I), Recovered (R), or Deceased (D)− and model transitions between these states over time (Fig. 1). These models use mathematical equations to represent underlying biological, physiological and social processes, providing a framework to explore pandemic scenarios and intervention impacts (Jit & Cook, 2024).

**Diagram flow of the SEIR/D model**: *The SEIR/D model divides the population into compartments representing disease states, with arrows depicting transitions based on specific rates. Here, the population is divided into the following compartments: Susceptible (i.e., individuals not immune and not yet exposed to the virus), Exposed (but not yet infectious), Infectious (individuals who can transmit the virus), Recovered or Deceased. Transitions between compartments are driven by parameters such as contact rate, transmission probability, latency, and recovery or mortality rates.*

The recent COVID-19 pandemic, caused by the Severe Acute Respiratory Syndrome Coronavirus 2 (SARS-CoV-2), highlighted the importance of advanced modelling approaches integrating social and behavioural factors, as they greatly influenced disease spread, containment, and public adherence to health measures (Bedson et al., 2021). COVID-19 is a respiratory disease transmitted between humans through four modes: direct physical contact between an infected and a susceptible individual, indirect contact with contaminated surfaces, large respiratory droplets, and fine aerosols (Leung, 2021). In the absence of vaccines or specific treatments in the pandemic's early stages, disease control relied mainly on NPIs. These interventions targeted different transmission modes at both individual (e.g., personal protective equipment, hand washing) and community levels (e.g., physical distancing) (Leung, 2021). Compartmental models can simulate the effect of these interventions by adjusting parameters, such as assigning lower contact rates to quarantined individuals compared to non-quarantined individuals (Kong et al., 2022). By modifying parameter values and compartmental structure, these models allow the exploration of pandemic evolution through forward simulations of various scenarios of NPIs implementation (e.g., the reduction in contacts due to quarantine or school closure) while accounting for uncertainties about multiple aspects of the disease itself (e.g., duration of infectiousness) (Holmdahl & Buckee, 2020). Additionally, they informed COVID-19 vaccine distribution strategies by emphasising age-based prioritisation to reduce mortality (Wagner et al., 2022). These models can also be used retrospectively and fitted to epidemic data to infer parameter values (e.g., to quantify and rank NPIs efficacy in real-world applications) (Flaxman et al., 2020). While these models have proven valuable, their accuracy is often constrained by underlying assumptions related to population heterogeneity.

### 1.2 Limitations of traditional infectious disease modelling

Traditional models frequently assume population homogeneity, ignoring real-world heterogeneities in transmission, susceptibility, and severity risks across population groups. Demographic factors such as age, gender/sex, and socioeconomic position influence contact rates, vulnerability, and exposure risks and should be incorporated to improve prediction accuracy and guide effective public health intervention (Gomes et al., 2024; Prem et al., 2017). Research indicates that models accounting for heterogeneities—such as age-related differences in contact patterns, protective behaviour, and physical distancing compliance—provided more accurate forecasts of disease spread (Bavel et al., 2020; Prem et al., 2017). Scholars increasingly advocate for integrating social and structural dimensions into infectious disease models, recognising that cultural norms, behavioural patterns, and social factors impact transmission dynamics (Bedson et al., 2021; Buckee et al., 2021; Manna et al., 2024). For example, culturally shared beliefs and norms within a population affect social behaviours and disease transmission. A study found that countries with predominant individualistic values—emphasising individual autonomy over collective responsibility—had lower adherence to protective measures, correlating with higher COVID-19 incidence and mortality (Naidoo et al., 2024).

### 1.3 Gender, sex, and COVID-19 epidemiology

Despite increasing recognition of demographic and social factors in disease transmission, gender and sex inclusion remains largely absent in models for respiratory diseases like COVID-19. This practice persists despite mounting evidence of gender and sex influences on transmission, disease susceptibility, severity, and mortality outcomes (Doerre & Doblhammer, 2022).

The COVID-19 pandemic highlighted the significant influence of gender and sex on disease epidemiology, with global studies reporting notable disparities in transmission patterns, incidence rates, severity, and mortality between women and men (Heidari & Goodman, 2021; Wenham et al., 2020). In many contexts, men were found to be tested less frequently but were more likely to test positive, more likely to be hospitalised, and had higher case-fatality ratios, leading to consistently higher mortality rates compared to women (Gebhard et al., 2020; Hawkes et al., 2022). These disparities reflect complex interactions between biological sex-related factors (e.g., hormonal and immune response variations) and gender-related social factors (e.g., occupational roles and health-related behaviours).

Integrating gender and sex in infectious diseases involves distinguishing between biological sex characteristics and gender-related social determinants while acknowledging their interrelated influence. Sex encompasses genetic, cellular, and physiological characteristics that typically differentiate females and males, influencing disease risk, pathology, morbidity, treatment response, and mortality (Amin et al., 2022). In infectious diseases, biological sex-based factors influence viral susceptibility, response to the virus, disease progression, and the effects and side effects of anti-infection or anti-inflammatory therapy (Meijs et al., 2022). Gender, as a social and structural determinant of health, encompasses societal roles, expectations, and norms that unequally allocate resources and opportunities among women, men, and gender-diverse groups, thereby shaping health outcomes (Risman, 2004). Gender influences health through five main pathways identified in Heise et al.’s theoretical framework: differential risk exposure, health-related behaviours, and healthcare access, along with gender-biased healthcare systems and health research further creating and reinforcing inequalities (Heise et al., 2019). Gendered dimensions of physical, social, and economic environments influence exposure, susceptibility, and severity risks for COVID-19. For instance, gender-based occupational segregation influences exposure risks, with women disproportionately represented in caregiving or service roles involving higher interpersonal contacts.

This paper aims to develop a conceptual framework that maps how gender and sex may influence COVID-19 transmission dynamics and outcomes within the SEIR/D model. By synthesising epidemiological and clinical findings, this paper identifies and characterises key mechanisms through which gender- and sex-related factors may affect transitions between compartments, providing a theoretical basis for their integration into infectious disease models. Additionally, this paper explores potential gender-related variations in surveillance data—such as in testing, hospitalisation, and mortality reporting—that may affect the calibration and validation of infectious disease models. Because these models often rely on surveillance data to estimate parameters and assess intervention strategies, understanding how gender-related disparities in healthcare-seeking behaviour, healthcare access and practices, and institutional protocols could influence data generation can provide insights to improve model accuracy and interpretation.

## 2 Methods

### 2.1 Literature search strategy

To investigate how gender and sex influence COVID-19 transmission dynamics and outcomes, a targeted narrative literature review was conducted, structured around the key processes represented by transitions within the SEIR/D compartmental model. This approach facilitated the identification of sex-related biological and gender-related social and structural factors potentially influencing disease progression and outcomes.

The literature search focused on peer-reviewed studies published in English between January 2020 and September 2024. It was primarily conducted using Google Scholar, which offers broad interdisciplinary coverage and facilitated the identification of relevant epidemiological, clinical, and modelling studies. In addition, PubMed was consulted to retrieve studies on sex-specific biological differences related to COVID-19 susceptibility to infection, severe outcomes, latency, asymptomaticity, and contagiousness, as described later in this section. The search strategy was structured around SEIR/D transitions, incorporating combinations of terms related to biological susceptibility, exposure risks, transmission dynamics, and health outcomes. These included: “gender”, “sex”, “women”, “men”, “females”, “males”, and “COVID-19”, “SARS-CoV-2”, with key relevant modelling components described below, such as “susceptibility to infection”, “occupational exposure”, “household transmission”, “non-pharmaceutical interventions”, “hospitalisations”, and “treatment efficacy”. As this was a narrative rather than a systematic review, searches were refined iteratively, and a snowball search strategy was employed by reviewing references cited in the selected studies to identify additional relevant literature. Where available, meta-analyses and systematic reviews were prioritised, as they synthesise findings across diverse research contexts.

The transition from Susceptible (S) to Exposed (E) is influenced by susceptibility to infection, contact patterns between susceptible and infectious individuals, environmental conditions, and the contagiousness of infectious individuals (Leung, 2021). The review first examined sex-related biological factors potentially affecting susceptibility. It then explored gendered exposure risks, focusing on how social roles, responsibilities, and occupational settings influence contact patterns. Particular attention was given to household and workplace settings, where gendered divisions of labour, caregiving roles, and occupational segregation likely influence exposure dynamics. The review also explored disparities in leisure and recreational activities, which were important drivers of superspreading events, as well as mobility patterns, which are frequently incorporated into mathematical models of disease transmission. Additionally, gendered disparities in adherence to non-pharmaceutical interventions (NPIs)—which could affect both exposure risk and transmission probabilities—were reviewed. Finally, the review examined sex-related biological factors influencing contagiousness.

For the transition from Exposed (E) to Infectious (I), the review focused on identifying potential sex-based differences in latency periods—the time from exposure to the onset of infectiousness. Additionally, given that some compartmental models differentiate between clinical and subclinical infectious states, the review assessed whether females and males had different probabilities of asymptomatic infection.

For the transition from Infectious (I) to Recovered (R) or Deceased (D), the review investigated sex-related biological factors influencing disease severity and susceptibility to severe outcomes. It also explored gender-related determinants affecting recovery and mortality, focusing on health-related behaviours, healthcare access, and potential gender biases in clinical practices and research, which may have influenced COVID-19 outcomes.

Finally, the review examined potential gender-related variations in epidemiological surveillance data, as infectious disease models often rely on such data for parameter estimation. Studies investigating disparities in testing uptake, hospitalisation rates, and mortality reporting were included to assess their potential impact on model calibration and interpretation.

## 3 Results: conceptual framework of gender and sex influence in SEIR/D models

### 3.1 Overview of gender- and sex-related factors in COVID-19 disease progression

**Fig. 2:**
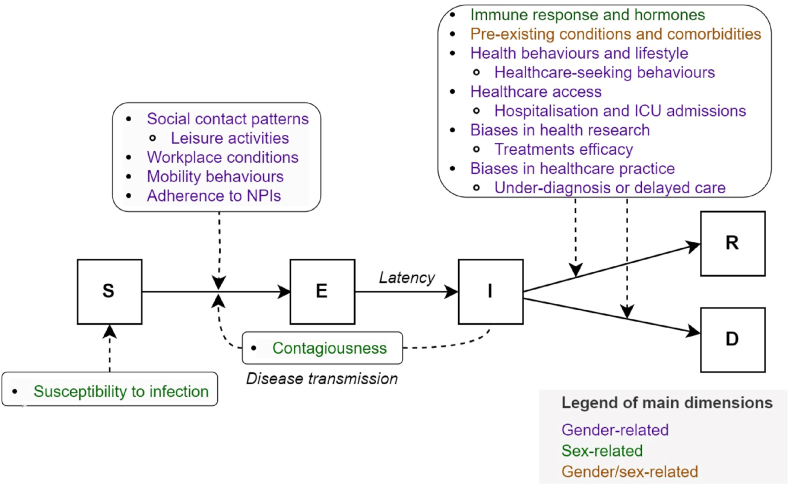
Synthesis of gender- and sex-related factors influencing transition across compartments **Notes:***Compartments include S=Susceptible, E=Exposed, I=Infectious*, *R=Recovered, D=Deceased. NPIs = Non-pharmaceutical interventions*. This figure synthesises findings from the literature review, categorising specific mechanisms by which gender-related (purple), sex-related (green), and combined (orange) factors may impact SEIR/D transitions.

The findings from the literature review are synthesised into the conceptual framework illustrated in Fig. 2, which maps gender- and sex-related mechanisms influencing transitions across SEIR/D compartments. This framework provides a structured visualisation of how gender- and sex-related factors may influence transition probabilities.

For the transition from Exposed (E) to Infectious (I) the literature search did not identify consistent variations between women and men, as meta-analyses reported no significant differences in incubation periods (Cheng et al., 2021) or asymptomatic infection rates (Sah et al., 2021). The specific factors involved are examined in the following subsections.

#### 3.1.1 Susceptibility

Biological differences between females and males may influence susceptibility to infection through variations in endocrine, metabolic, and immune functions (Bechmann et al., 2022). Males’ distinct immune responses may be influenced by higher expression of angiotensin-converting enzyme 2 (ACE2), the primary receptor for SARS-CoV-2 entry. Conversely, females may benefit from stronger immune activation due to genetic factors, such as the presence of two X chromosomes, and hormonal profiles, particularly oestrogen, which is associated with enhanced immune responses (Bechmann et al., 2022; Gebhard et al., 2020; Scully et al., 2021; Takahashi et al., 2020).

#### 3.1.2 Exposure

Exposure risk to SARS-CoV-2 is primarily influenced by social and environmental factors, as transmission predominantly occurs during physical interactions. These factors are closely linked to gender, as differential exposures to health risks between women and men are mainly stemming from their socially ascribed roles (Heise et al., 2019). Transmission risks are amplified by direct and prolonged contact, making environments where people are in proximity for extended periods–such as households and workplaces–high-risk settings. Exposure levels are influenced by social contact patterns, work conditions, mobility behaviours, and adherence to NPIs during crises (Manna et al., 2024).

The first dimension of gendered exposure factors relates to social contact patterns, which vary importantly depending on the setting and are affected by the division of labour and social roles. Studies examining social contact patterns often differentiate between household, workplace, and educational environments. Households are identified as the highest risk setting for transmission, followed by workplaces (Manna et al., 2024). Research conducted in high-income countries, which pooled data from several studies, found no significant difference in overall contact rates between women and men (Mousa et al., 2021). However, contact locations differed: women had a higher proportion of daily contacts at home, while men had more contacts at work. This pattern aligns with traditional gender roles, with men more engaged in the labour market and women more involved in domestic and caregiving activities (Risman et al., 2018). Evidence supporting the influence of these social roles and the underlying gender division of labour comes from several studies. For instance, research conducted in China demonstrated that women were more likely to be infected within the family, whereas men were more likely to be the external source of the family cluster (Liao et al., 2023). Similarly, a retrospective cohort study in Spain found that women had a higher risk of contracting SARS-CoV-2 at home and were more likely to be infected by a cohabiting infected individual compared to men (Soriano López et al., 2024).

Based on these findings, we identified several gender-related factors influencing exposure risks within the place of residence: (1) Household contacts, characterised by proximity and extended interactions, pose higher exposure risks for women compared to men and are potentially the primary site of exposure for them; (2) Gender differences in living arrangements considering the life course may lead to differential exposure risks – women are more often primary caregivers in single-parent families, and children serve as important vectors for COVID-19 transmission (Prem et al., 2017; Soriano López et al., 2024). In addition, women's caregiving roles as primary caregivers for children and older adults likely increase their exposure risks, especially when caring for sick relatives; (3) Gender disparities in living conditions also affect exposure. Men are overrepresented in crowded communal settings such as prisons, homeless shelters, and military barracks, where maintaining physical distancing and following health recommendations is challenging, increasing transmission risks (Hagan, 2021).

Workplace exposure is another central domain of gendered exposure risks. Social interactions vary by occupation, with healthcare practitioners, personal care workers, and service providers generally facing elevated exposure to contagion (Lewandowski et al., 2021). In Europe and other high-income countries, women are disproportionately represented in sectors requiring direct contact with people, such as healthcare and education, and comprise the majority of low-paid “essential workers” (i.e. system-relevant workers) (Doerre & Doblhammer, 2022; Lewandowski et al., 2021). This occupational segregation often results in women facing higher contact-related risks. While women more often work in high-contact healthcare or education sectors, protective measures may be more strictly enforced, especially in healthcare settings. In contrast, men-prevalent sectors such as trades, transport, and certain professional occupations showed a lower likelihood of enforcing protective measures. A UK study found that workers in these sectors had reduced access to protective equipment, such as face masks, and were less likely to practice frequent hand hygiene (Beale et al., 2023), contributing to gendered infection risks. In the US, a high number of cases and deaths were reported early in the pandemic among workers in the meat and poultry processing industry, where men comprise three-quarters of the workforce (Data USA, n.d.). These environments – characterised by close physical proximity, shared equipment, poor hygiene practices, and suboptimal ventilation – facilitated the spread of the virus (Dyal et al., 2020). Indeed, certain low-skilled or low-paid occupations, where men make up the majority of workers (e.g., food processing, transportation, delivery, warehousing, construction, manufacturing), were associated with higher mortality risks due to greater exposure and exacerbated social vulnerability (Griffith et al., 2020). Patterns of working from home seemed also gendered. Pre-pandemic data indicated that women were more likely to work from home, and during the COVID-19 pandemic, data from Europe and the US showed a significant increase in working from home for both women and men, though women experienced higher rates (Touzet, 2023), potentially offering them greater viral protection. Gender division of labour often places men more frequently in public settings (e.g., social gatherings, recreational activities, and work-related travel), potentially elevating their exposure risk outside of the home.

Leisure and recreational activities have played an important role in superspreading events, with gendered patterns influencing exposure risks. Although few studies have adopted a gender perspective within the context of COVID-19, a Brazilian study demonstrated that men were more likely to engage in collective sports, such as basketball and football, compared to women, who showed a greater interest in indoor artistic and manual activities like cooking and handicrafts (Bridi et al., 2023). Activities such as singing, which involve high levels of droplet emission, presented particularly high transmission risks (Bahl et al., 2020); these activities are more commonly practised by women (Parkinson, 2019), illustrating the complexity of gendered patterns in leisure activities and their impact on COVID-19 exposure risks.

Mobility patterns also influence exposure risks. Women are more likely to rely on public transport for caregiving duties, domestic errands, and commuting to work or driving relatives, while men's transportation is more often associated with individual car use, typically for professional purposes (Bambra et al., 2021; Parnell et al., 2022). Women were also more likely to reduce their mobility during the COVID-19 lockdown than men, who were more likely to frequently visit recreational areas (Bulteau et al., 2023; Reisch et al., 2021). The gender gap in mobility was particularly pronounced among working and reproductive-age groups, reflecting the gendered division of unpaid and paid labour often observed among heterosexual couples—specifically, the gender-specific organisation of household responsibilities and work patterns (Reisch et al., 2021).

#### 3.1.3 Non-pharmaceutical interventions (NPIs) adherence

Gender disparities in adherence to NPIs constitute important mechanisms influencing exposure risk. Consistent evidence shows that women were more likely to adhere strictly to SARS-CoV-2 preventive measures and hold more favourable COVID-19−related beliefs and attitudes compared to men. These disparities are attributed to factors such as risk perception (Galasso et al., 2020), trust in government (Nivette et al., 2021), and conformity to gender norms (Jones & Anderson, 2024; Mahalik et al., 2022a).

Men's lower adherence to NPIs may be influenced by dominant masculine norms that discourage expressions of vulnerability and emphasise self-reliance and risk-taking (Mahalik et al., 2022b). A US study found that sexist beliefs, correlated with dominant masculine norms, were associated with lower COVID-19 risk perception, reduced protective behaviours, less support for containment policies, and higher infection likelihood (Reny, 2020). Conversely, men with more egalitarian gender role beliefs showed higher compliance (Paramita et al., 2021). This could potentially contribute to higher transmission rates in men-prevalent environments, particularly in settings where NPIs enforcement is less rigorous. Another study found that the relationship between attitudes toward mask-wearing and conformity to masculinity norms was mediated by perceived benefits and barriers, confidence in the scientific community, and empathy toward vulnerable persons (Mahalik et al., 2022b).

#### 3.1.4 Contagiousness

The contagiousness of infected individuals is a key determinant of transmission dynamics, with viral load as a major factor. Higher viral loads correlate with an increased concentration of virus in respiratory secretions, potentially enhancing the probability of transmission upon contact. Several studies have reported higher viral loads among males compared to females (Bechmann et al., 2022; Herbert et al., 2023). This finding suggests that males may more readily transmit the infection, potentially leading to a higher number of secondary cases originating from male index patients and an increased overall risk of transmission in male-majority environments. However, the relationship between viral load and sex, as well as age dynamics, are highly debated (Puhach et al., 2023), highlighting the need for further research to provide conclusive evidence.

#### 3.1.5 Probability of recovery and death

The progression of SARS-CoV-2 infection, influencing recovery and mortality outcomes, is influenced by a combination of infection severity, biological vulnerability, and gendered social determinants of health. The sex-related biological mechanisms discussed below are among the most commonly cited explanations for the greater severity of COVID-19 observed in males; however, additional mechanisms are detailed in comprehensive reviews (Aksoyalp & Nemutlu-Samur, 2021; Gay et al., 2021; Gebhard et al., 2020; Taslem Mourosi et al., 2022).

Takahashi et al. have described that sex-linked factors influencing recovery and death are related to the immune response and modulation (Takahashi et al., 2020). An important sex-linked factor influencing recovery and death is the difference in immune responses between females and males. Females generally exhibit stronger immune responses, potentially leading to faster recovery and reduced mortality risk from COVID-19. Studies have indicated that males often demonstrate higher levels of certain pro-inflammatory cytokines, such as IL-8 and IL-18, during SARS-CoV-2 infection. While these cytokines are components of the innate immune response, their elevated levels can trigger excessive inflammation, known as a cytokine storm, which is associated with severe COVID-19 outcomes and increased mortality rates in males. In contrast, stronger T-cell activation in females may lead to more effective viral control and a milder disease course. These immune variations contribute to a greater likelihood of recovery and lower mortality risk among females compared to males (Klein & Flanagan, 2016).

The role of sex hormones in immune modulation is another key factor. Oestrogen, predominant in females, enhances both innate and adaptive immune responses, potentially facilitating more efficient viral clearance and reducing the risk of severe outcomes. Additionally, oestrogen modulates inflammatory response, potentially mitigating cytokine storm risks. Conversely, testosterone, present in higher levels in males, has been linked to immunosuppressive effects that may lead to poorer outcomes. Genetic differences related to the X chromosome, which carries numerous immune-related genes, present another potential factor. Females, with two X chromosomes, may benefit from a double occurrence of these genes, potentially conferring an immunological advantage that contributes to more effective viral clearance and reducing mortality risk.

Gender and sex differences in pre-existing conditions and comorbidities are thought to be key contributors to the higher prevalence of severe outcomes observed in men (Vahidy et al., 2021). These differences arise from both biological factors (genetic and hormonal influences) and gendered social conditions that favour comorbidity development and progression. Men have a higher prevalence of conditions such as cardiovascular disease, hypertension, diabetes, and chronic lung disease, which elevate the risk of severe COVID-19 outcomes (Bechmann et al., 2022). The higher prevalence of risk factors among men, such as smoking and alcohol consumption, is shaped by gender norms influencing lifestyle choices. These factors may exert both direct effects (e.g., increased ACE2 expression in the lungs, providing more entry points for the virus) and indirect effects (e.g., weakened immune function) on disease severity (Bechmann et al., 2022).

Gendered disparities in healthcare access may further impact treatment and recovery outcomes. A study on nursing home residents, a population predominantly composed of women, revealed declining hospitalisation rates during the pandemic (Maxwell et al., 2024), raising concerns about both gender-based access disparities and the gendered effects of healthcare system organisation. This finding aligns with research indicating significantly lower hospital and ICU admissions for women during the first wave in Spain, with adjusted odds ratios of 0.44 and 0.37, respectively (Aguilar-Palacio et al., 2024). These disparities may reflect gender biases in healthcare access, disadvantaging women through differential triage or referral practices. Once hospitalised, however, females demonstrated higher survival rates in ICUs compared to males, even after controlling for age, risky behaviours, comorbidities, disease severity and treatment received (Meijs et al., 2022). This suggests that sex-specific biological mechanisms, such as immunological and hormonal variations, may confer a recovery advantage for females. However, statistical adjustments for these factors may inadvertently obscure their distinct contributions to survival outcomes between females and males.

Another gender-related challenge is the knowledge gap concerning differences in COVID-19 treatment efficacy, a gap linked to historical androcentric biases in health research (Heise et al., 2019). A review of 4420 registered SARS-CoV-2 clinical studies on ClinicalTrials.gov found that only 4 % planned to consider sex/gender as an analytical variable, and only a minority of published trials reported sex-disaggregated outcomes (Brady et al., 2021). Similarly, a scoping review noted the absence of gender/sex-stratified analyses in early COVID-19 clinical trials for pharmacological therapies (Schiffer et al., 2020). Limited existing research suggests sex differences in the effectiveness of treatments such as remdesivir and colchicine (Aksoyalp & Nemutlu-Samur, 2021).

Potential gender bias in clinical decision-making may differentially affect the quality and probability of care received by women and men. A study of COVID-19 clinical case reports found a bias in reporting male patients and in the authorship of these reports, with male authors more likely to describe male patients (Salter-Volz et al., 2021). Such biases may have contributed to disparities in care and unequal dissemination of clinical information, especially given the absence of sex-specific guidelines for managing COVID-19. Gender/sex differences in symptom presentation between women and men have also been reported (Heidari, 2022). This variation may lead to underdiagnosis or misclassification of COVID-19 in women, particularly if healthcare providers are not trained to recognise the full spectrum of sex-specific symptoms, potentially affecting patient outcomes.

#### 3.1.6 Surveillance data and reporting

Models are generally fitted to surveillance data, which may reflect gendered patterns in reporting and healthcare access, influencing case detection, hospitalisation, and mortality statistics. These potential patterns should be carefully considered to ensure an accurate interpretation of disease dynamics. Global data indicates that, with women as the reference group, the gender/sex ratio in the COVID-19 care cascade was 0.81 for testing, 1.21 for hospitalisations, 1.91 for ICU admissions, and 1.28 for mortality (Hawkes et al., 2022).

Globally, women have been found to undergo SARS-CoV-2 testing more frequently than men, potentially driven by several factors. These include greater adherence to health recommendations (Galasso et al., 2020), more proactive health-seeking behaviours (Paramita et al., 2021), and higher healthcare services utilisation (Simons et al., 2023). Women's caregiving roles may also contribute, as testing decisions may serve as a symbolic act of care and protection for others (Nørholm et al., 2024). Potential sex differences in symptom manifestation due to females' generally stronger innate and adaptive immune responses to infection (Massion et al., 2023) and gendered tendencies in symptom recognition – with women more likely to report symptoms (Ladwig et al., 2000) – may further influence testing uptake. Finally, women may have been tested more frequently, due to their overrepresentation in healthcare professions, a sector often subject to routine testing protocols (Danielsen et al., 2022). Importantly, these disparities fluctuated throughout the pandemic, potentially influenced by evolving epidemiological situations as well as differing testing capacities and strategies over time. These factors may result in a higher proportion of women in case reporting data, as testing rates directly influence the likelihood of cases being identified and reported.

Hospitalisation data must also be interpreted within the broader healthcare system's organisation, which may reflect gendered patterns in care access. Women, who constitute the majority of nursing home residents, may be less likely to be hospitalised due to protocols prioritising on-site treatment or limiting access to external healthcare facilities. This dynamic may contribute to the lower hospitalisation and ICU admission rates observed in women and could obscure unmet healthcare needs among them, particularly for severe COVID-19 cases, as men were twice as likely to be admitted to the ICU (Hawkes et al., 2022).

Mortality data may also reflect gendered reporting disparities. Women's longer life expectancy increases their likelihood of dying outside hospital settings, where SARS-CoV-2 testing may not consistently occur. A comparative study of 32 countries showed that the increase in at-home deaths between 2018–2019 and 2020–2021 was higher in women than in men (Lopes et al., 2024), potentially affecting the accuracy of COVID-19 mortality statistics. This pattern may lead to an underreporting of COVID-19-related deaths among women.

## 4 Discussion

This paper presents a conceptual framework that maps the multifaceted influence of gender- and sex-related factors on COVID-19 transmission dynamics and outcomes, synthesising epidemiological and clinical evidence. Integrating these factors into compartmental models requires refinements in structure, parameter estimation, and calibration to better account for population heterogeneity. For instance, creating separate mortality compartments for women and men could improve predictions of healthcare demand based on sex-specific severity risks. Additionally, incorporating gender-specific exposure risks and NPIs adherence could refine transmission estimates and ensure interventions do not disproportionately benefit or disadvantage specific groups (Wenham et al., 2020).

While this paper used an SEIR/D framework to guide conceptualisation, its conclusions also apply to Agent-Based Models (ABMs) as well, even more so if we consider that ABMs address key limitations of compartmental models, particularly the assumption of homogenous mixing and the difficulty of capturing adaptative behavioural changes. ABMs offer greater flexibility by modelling individual heterogeneity and dynamic social interactions, allowing for a more detailed representation of behavioural adaptations in response to disease spread and policy interventions. However, their high level of complexity and their reliance on granular individual-level data, which is often unavailable, pose challenges for scalability and model calibration (Naidoo et al., 2024). The choice of modelling framework should be guided by data availability and modelling objectives, as both approaches have distinct strengths and limitations.

This paper also highlights the added value of considering structural gendered settings and behaviours in modelling, moving beyond individual gender identity to account for the multidimensional and context-dependent nature of gender as a social and structural determinant of health (Göttgens & Oertelt-Prigione, 2023). By focusing on underlying mechanisms— such as gendered occupational exposure and social norms shaping health behaviours—modelling could better reflect how gender influences disease transmission and outcomes. For example, tailoring communication campaigns to address lower NPIs adherence among men (Mahalik et al., 2022b) or adapting occupational health policies for gender-specific workplace risks could mitigate disparities and promote public health equity.

The conceptual framework primarily focuses on women/females and men/males’ groups, reflecting data limitations and the predominance of binary indicators in epidemiological and clinical research. Future studies should expand this conceptual analysis to gender-diverse populations, which remain marginalised in health research and surveillance data collection. Additionally, the review highlights that binary classification is a simplification that does not fully capture the independant and interacting influences of gender and sex on health outcomes. For example, variations within groups—such as differences in adherence to NPIs among men with traditional versus progressive views (Paramita et al., 2021)—illustrate the limitations of binary categorisation in accounting for gendered behavioural patterns. Similarly, following Pape et al.'s argumentation, intra-group hormonal variations may contribute to COVID-19 severity outcomes in ways that binary sex categories alone do not fully capture (Pape et al., 2024). While binary stratification remains common in epidemiological practice, alternative stratifications—such as occupation type, caregiving responsibilities, risk perception, or hormonal profile—could provide a more precise representation of disease transmission and severity. However, collecting variables linked to occupation, social behaviours, or hormonal levels remains challenging in real-time epidemic response and routine surveillance.

Pandemics tend to exacerbate pre-existing social inequalities, creating feedback interactions that influence COVID-19 outcomes. For instance, gendered caregiving responsibilities and economic disparities were further intensified by increased unpaid care burdens and disproportionate job losses among women during the pandemic (Flor et al., 2022), which not only elevated exposure risks but also potentially limited access to healthcare, indirectly worsening health outcomes. These dynamics underscore the importance of adaptive modelling approaches that incorporate evolving gendered risk patterns over time (Danielsen et al., 2022; Viguerie et al., 2020).

While this paper aims to provide a comprehensive synthesis, the narrative review approach may have overlooked certain gender- and sex-related factors. Moreover, as this was not a systematic review, the selection of studies was guided by conceptual relevance rather than predefined inclusion criteria, which limits replicability. Additionally, this review did not systematically examine the intersections of gender and sex with other structural and social determinants, as this was beyond its scope but warrants further exploration. For example, age influences contact patterns (Prem et al., 2017), while socioeconomic position affects exposure risks (Khalatbari-Soltani et al., 2020) and contributes to gendered mortality disparities (Auderset et al., 2024).

This paper introduces theoretical considerations to stimulate further research on gender- and sex-sensitive modelling approaches. Locally tailored models and region-specific interventions are necessary to reflect the variability of gender norms and their health impacts across cultural and geographic contexts (Heise et al., 2019). For example, gendered patterns of occupational exposure, shaped by cultural and institutional factors (Lewandowski et al., 2021), require context-specific investigation for accurate integration. More broadly, variations in epidemiological conditions, healthcare system organisation, and the implementation of NPIs influence disparities in COVID-19 outcomes between women and men (Danielsen et al., 2022; Sant Fruchtman et al., 2022). Moreover, as disease transmission models often rely on surveillance data, the potential for gender-related variations in case detection, hospitalisation rates, and mortality statistics should be considered. Higher testing uptake among women could inflate their case representation and skew infection rate estimates, while lower hospitalisation rates among women could also reflect unmet healthcare needs rather than differences in disease severity alone. Accounting for these variations could improve the robustness of model outputs and inform more effective and equitable policy decisions.

Our approach systematically considered gender and sex dimensions at each stage of the SEIR/D model, identifying their potential influence on key transition probabilities. However, translating these mechanisms into quantifiable estimates–beyond the scope of this conceptual framework–presents challenges. The identified factors are not mutually exclusive and can affect outcomes and transmission dynamics in complex and occasionally conflicting ways, potentially neutralising their effects at the population level. Despite these challenges, delineating these mechanisms paves the way for advancing gender- and sex-sensitive modelling approaches. Addressing these challenges requires interdisciplinary collaboration between modellers, social scientists, and clinicians to translate social and biological processes into actionable model inputs (Bedson et al., 2021). Future research should systematically incorporate gender- and sex-based perspectives into epidemiological and clinical studies to clearly delineate the differential risk patterns for infectious diseases like COVID-19 and clarify the mechanisms driving disparities (Palmer-Ross et al., 2021). The current lack of these systematic perspectives in this area limits the development of robust and context-sensitive models (Brady et al., 2021; Doerre & Doblhammer, 2022).

In conclusion, integrating gender and sex considerations into infectious disease models offers the potential to enhance predictive accuracy, inform tailored interventions, and address health inequities. This paper develops a conceptual SEIR/D framework that maps the influence of gender- and sex-related factors on COVID-19 transmission dynamics and outcomes. Moving forward, efforts should focus on empirically validating these mechanisms, developing alternative stratifications, and fostering interdisciplinary collaboration to advance the mathematical modelling of infectious diseases.

## Funding

This work was supported by the University of Lausanne [Mobi.Doc scholarship, ID number MD-0063 and ProFemmes scholarship].

## CRediT authorship contribution statement

**Diane Auderset:** Writing – review & editing, Writing – original draft, Visualization, Methodology, Investigation, Funding acquisition, Conceptualization. **Julien Riou:** Writing – review & editing, Validation, Supervision, Conceptualization. **Carole Clair:** Writing – review & editing, Validation, Funding acquisition. **Matthieu Perreau:** Writing – review & editing, Validation. **Yolanda Mueller:** Writing – review & editing, Validation, Supervision, Funding acquisition, Conceptualization. **Joëlle Schwarz:** Writing – review & editing, Validation, Supervision, Funding acquisition, Conceptualization.

## Ethical statement

Ethics approval was not required for this study as it is based on a review and synthesis of previously published literature. All sources have been appropriately cited to ensure transparency and acknowledgment of original contributions.

## Declaration of generative AI in the writing process

During the preparation of this work, the first author used ChatGPT (Scholar) and Claude (Sonnet 3.5 version) as redactional assistants in order to refine the linguistic aspects of the manuscript. All suggestions for improvement were carefully reviewed and selectively implemented. The author reviewed and edited the content as needed and takes full responsibility for the content of the article.

## Declaration of interest

The authors declare that they have no known competing financial interests or personal relationships that could have appeared to influence the work reported in this paper.

## Data Availability

No data was used for the research described in the article.
